# Reach, Adoption, and Implementation Strategies of a Telehealth Fall Prevention Program: Perspectives From Francophone Communities Across Canada

**DOI:** 10.1177/15248399241252807

**Published:** 2024-05-17

**Authors:** Jennifer O’Neil, Nathalie Dionne, Sylvie Marchand, Dominique Cardinal, Grant Handrigan, Jacinthe Savard

**Affiliations:** 1 Université d’Ottawa, Ottawa, ON, Canada; 2 Institut de Recherche Bruyère, Ottawa, Ontario, Canada; 3 CNFS-Volet Université d’Ottawa, Ottawa, Ontario, Canada; 4 Universtité de Moncton, Moncton, New Brunswick, Canada

**Keywords:** access to health care, technology, aging, community intervention, health promotion

## Abstract

*Introduction*. A fall may impact a person’s physical, emotional, and psychological well-being. Fall prevention programs are being implemented to reduce these negative outcomes. However, linguistic barriers in health services may reduce access to such prevention programs. A telehealth fall prevention program was designed to increase access to such programs in French for Francophone minority communities in Canada. This capacity-building project aimed to support community partners to deliver this telehealth program and document strategies used to reach, adopt, and implement the program within various Francophone and Acadian Minority Communities. *Methods*. A sequential explanatory mixed methodology was used to document reach, adoption, and implementation strategies and describe the lived experiences of program facilitators and organization representatives. Reach, adoption, and implementation were documented and analyzed descriptively, while lived experiences were analyzed using content analysis following the Consortium Framework for Implementation Research. *Results*. Twelve organization representatives or program facilitators from eight organizations operating in four different provinces participated in the study. Three themes emerged from the qualitative data on reach and adoption: external context, internal context, and capacity building. Four themes were identified as barriers and facilitators to implementation: level of preparation and time management, interpersonal relations and telepresence, exercise facilitation and safety, and technological problem-solving. *Conclusion*. Using tailored reach and adoption strategies such as prioritizing provinces with higher proportions of needs and training local community program facilitators may lead to the successful implementation of a new telehealth fall prevention program. Results from this study could potentially inform other primary prevention programs or telehealth program implementation.

A fall can lead to a significant injury which may impact a person’s physical, emotional, and psychological well-being. Falls in older adults are the leading cause of injury or emergency visits in Canada. Current evidence around multifactorial fall prevention programs that focus on people presenting in the emergency department due to a fall does not demonstrate a significant impact on fall incidence (Morello et al., 2019). Therefore, the implementation of programs for people at risk of falls before they need medical care should be further explored.

Linguistic barriers in health services may lead to delaying/reducing preventive consultations, poor health outcomes, and poor health service quality (Bowen, 2001, 2015; de Moissac & Bowen, 2019). In fact, a report published in 2019 on aging francophones in Ontario suggests that overall health indicators are impacted by linguistic preferences (Assemblée de la francophonie de Ontario, 2019). Since health services offered in English for francophone older adults may result in increased stress, reduced comprehension, and overall confidence in their care (Bouchard et al., 2023), offering health interventions in French for francophones may improve therapeutic relationship (Drolet et al., 2015) and overall intervention effectiveness. Therefore, implementing fall prevention programs tailored to older adults in their language of choice may be a solution to reduce falls among francophone older adults living in minority situations. Telehealth increases the potential to reach small or remote communities in which local French-speaking health care professionals are not available.

With an overarching goal of improving access to health services in French, the telehealth fall prevention program *Marche vers le futur (MVF)* is an effective primary fall prevention program prioritizing francophone adults 55 years and older, who present with fall risk factors (Savard et al., 2018). This 12-week multifactorial program including 2 weeks of assessments and a 10-week intervention was developed for francophone and Acadian Minority Communities (FAMC).

Implementation literature reports several factors capable of positively or negatively influencing the implementation success of a new health program within a health optimization approach. These are the presence of local leaders, the social influence, and local context (Goorts et al., 2021). However, there is limited research around implementation strategies in the context of health primary prevention in FAMC or regarding telehealth programs that incorporate multifactorial components (i.e., education, exercises, and discussions). This study will provide information on lived experiences and the strategies used by francophone organization representatives and program facilitators to reach communities and improve adoption, leading to the successful implementation of the MVF program across FAMC. This study aims to document (1) reach—defined as the potential individuals and eligible communities, (2) adoption—focusing on internal and external contexts and reasons for adoption, and (3) implementation—perspectives from program facilitators.

## Methodology

A sequential explanatory mixed method was used to document the strategies used by organization representatives and program facilitators and to understand the lived experiences of implementing the telehealth MVF program. The conceptual frameworks RE-AIM (Kwan et al., 2019) and its extension PRISM (McCreight et al., 2019; Trinkley et al., 2020) were used to guide data collection. The Consortium Framework for Implementation Research (CFIR) (Damschroder et al., 2009) was used to inform data analysis. External validity was established by using the RE-AIM checklist to report information on reach and adoption. The Good Reporting of A Mixed-Method Study (GRAMMS) checklist as well as the six recommendations to improve adequate reporting of the methodology (Lengnick-Hall et al., 2022; O’Cathain et al., 2008) was also used to enhance rigor in reporting on implementation.

### Participants and Sampling

Purposeful sampling was used to recruit organization representatives and program facilitators in FAMC interested in providing health prevention services to francophone older adults in their communities. Each organization representative or program facilitator who adopted the program was invited to participate in the study and signed a consent form prior to completing any questionnaires.

### Intervention

The MVF fall prevention program delivered remotely is tailored and effective in reducing fall risk factors in francophone adults aged 55 and over. Details of the program can be found in Savard et al. (2018) and in Appendix. Each 90-minute session of the intervention offered by a trained program facilitator once a week, for 10 weeks includes an educational component and discussion around fall risk factors followed by an exercise program targeting balance, strength, and flexibility. The facilitators of the MVF program are now required to follow a 2-day training session prior to the implementation phase and a 3-in-session follow-up during implementation. In this study, funding was available for the community agency who wanted to implement the project. Two versions of the program were offered: MVF community where participants met in a local center to complete the program and MVF home where participants connected virtually from their residence.

### Demographic and Quantitative Data

Demographic data including gender identity, language preferences, age, and professional experience were used to contextualize the group of facilitators and organization representatives. Reach, adoption, and implementation data were documented before, during, and after program implementation. Reach was measured as the number of the francophone community contacted by the team, the number of provinces involved, and the number of program facilitators recruited. Adoption was documented by the number and percentage of communities implementing the program, and the setting of each community. Implementation was documented descriptively by documenting the number of participants who completed the assessments, who participated in the program, as well as attrition. Representatives’ and program facilitators’ perspectives on the implementation were also documented.

### Qualitative Data

Data reporting on lived experiences related to the reach of organization representatives and program facilitators and adoption of the program were documented using individual interviews. Implementation of the program was documented using field notes. An MVF trainer (ND) attended the first, second, and sixth sessions in person or virtually to document program implementation through field notes for each community.

### Data Analysis

A mixed method of analysis was used to triangulate the data. Demographic data were analyzed descriptively (i.e., percentage, mean, and standard deviation). Quantitative data were categorized between reach and adoption and analyzed descriptively (i.e., percentage and mean). Pre-program implementation perspectives were compared with post-program results using the CFIR framework and using implementation outcomes to document if implementation expectations were under- or overestimated.

Qualitative data were analyzed using content analysis following the CFIR framework. Field notes were deductively organized by types of strategies (i.e., reach, adoption, or implementation). Furthermore, the field notes documenting the progress of the program facilitators over the 10 weeks program implementation were deductively categorized under barriers or facilitators. Data from the interviews was transcribed and inductively coded into new themes by two physiotherapist-researchers involved in the implementation of the MVF. The lived experiences of the program facilitators and organization leaders were reviewed by the research team including occupational therapists, physiotherapists, and MVF program developers. The quantitative and qualitative data were then integrated in the discussion to allow contextualization of the information.

## Quantitative Results

### Demography

Eight organizations from 4 provinces, 3 organization representatives, and 10 program facilitators consented to participate in this study. One organization representative dropped out due to family reasons. Of the 12 organization representatives or program facilitators who completed the study, most were francophone females born in Canada and identifying as cisgender ranging between 20 and 59 years old without previous experience implementing a telehealth program. Five health professions were represented and included physiotherapy (25%), occupational therapy (25%), kinesiology (17%), physical activity or specialized education (17%), social work (8%), and retired health care workers (8%) (Table 1). Together, they implemented 16 MVF programs, representing one to three implementations per community.

**Table 1:** Organization Representatives and Program Facilitators’ Characteristics

Characteristics at the individual level	Percentage (%)
**Sex**	
Female	75
Male	25
**Gender identity**	
Cisgender	100
**Age**	
20–29	42
30–39	0
40–49	17
50–59	25
60–69	17
70+	0
**Maternal language**	
French	83
English	17
**Preferred language**	
French	92
English	8
**Born in Canada**	
Yes	100
**Health professions represented**	
Physiotherapy	25
Occupational therapy	25
Kinesiology	17
Physical activity or specialized education	17
Social work	8
Other	8
**Previous facilitation via telehealth**	
Yes	25
No	75

### Setting Characteristics: Reach

Thirty-four francophone communities were contacted between June 2021 and September 2022, using three communication modalities such as email, phone calls, and online meetings, for an average of 5–10 hours per week allocated to relationship building. Reach and recruitment efforts were also supported by a promotional video and an infographic presenting the program. Each community was contacted by the same person multiple times by email, followed by a phone call or online meetings. Phone calls or online meetings ranged between 20 and 60 minutes per community. The median age of the francophone population in the contacted provinces who responded (NB, AB, ON, NS, PEI, Nunavut, YK, and TNO) ranged between 37 and 55.2 years old, and the percentage of individuals declaring French as their first official language spoken ranged between 0.5% and 31.8% (Table 2).

**Table 2:** Characteristics of Contacted Communities (i.e., Percentage [%] of Francophones Living in the Community, Median Age of the Francophone Adults, Percentage of Older Francophone Adults, and Rural–Urban Proportions). Characteristics of Community Who Adopted the Program MVF Are in Bold

Francophone communities in each province-territories	Francophones living in province (%)	Median age of francophone adults (years)	Proportion of older francophone adults (60 years and over) (%)	Proportion of francophones living in rural/urban areas (%)
British Columbia	1.4	51.1	30,8	13.2/86.8
**Alberta**	**2**	**43.9**	**21,7**	**16.1/83.9**
Manitoba	3.2	51.4	32,8	27.8/72.2
Saskatchewan	1.3	55.2	39	35.1/64.9
**Ontario**	**4.1**	**47.9**	**27,1**	**17.8/82.2**
**New Brunswick**	**31.8**	**48.8**	**29,9**	**51/49**
Nova Scotia	3.2	53.8	36,9	58.5/41.5
**Prince Edward Island**	**3.3**	**52.4**	**34,1**	**55.3/44.7**
Yukon	4.6	41.1	16,8	17.6/82.4
Northwest Territories	3	37.5	9,7	100/0
Newfoundland and Labrador	0.5	45.4	25,9	45.8/54.2
Nunavut	1.8	37	10,5	—
Total francophones outside Quebec	3.8	48.4	28.1	27.7/72.9

*Note.* These statistics were taken from the Government of Canada, Canadian Heritage Official, 2016 Census of Canada, Statistics Canada-Language Minority Communities Dashboard (2021).

### Setting Characteristics: Adoption

Out of 34 communities contacted over 1 year, 8 organizations from 4 different provinces adopted the telehealth program (24%). Hospitals, community health centers, and community programs, respectively, represented 13%, 25%, and 63% of the settings in which the program was adopted. Overall, 33 program facilitators were trained over 10 2-day courses between December 2021 and February 2023. Of the provinces which implemented the program, one facilitator was trained in Alberta, six in New Brunswick, four in Prince-Edward Island, and eight in Ontario.

### Setting Characteristics: Implementation

A total of 146 older adults were assessed and 129 completed the program (88%). On average, 2.4% attrition was documented (*n* = 7) following the initial assessment.

### Pre-Implementation Perspectives From Program Facilitators Compared With the Reality

Program facilitators who responded to the preprogram questionnaire (*n* = 7) slightly underestimated the potential recruitment of older adults, estimating 14 participants (±3.14) compared with the reality which averaged 18 participants per program. As for the percentage of older adults accepted into the program, facilitators underestimated the acceptance rate in the preprogram questionnaire, estimating 82.9% compared with the reality with 93% of older adults accepted. Regarding the type of telehealth service provided, program facilitators accurately anticipated the location of the MVF telehealth program, estimating 80.7% of the program delivery in centers and 22.1% at home compared with an actual 80.8% and 19.2%, respectively.

## Qualitative Perspectives From Community Representatives and Program Facilitators

### Reach and Adoption

Reach and adoption data were categorized into external context, internal context, and capacity building. Three sub-themes emerged in the external context category: pandemic-related barriers, infrastructure, and regional priorities. Community representatives and program facilitators reported that public health restrictions caused delays in adopting the new program. This was mostly related to limited human resources including limited francophone health care professionals capable and willing to facilitate the MVF program. Program facilitators and representatives shared that the MVF program allowed them to reduce isolation, offer a service in French, increase physical activity in older adults, and fulfill a gap around fall prevention francophone programming in their region. Technology literacy was a limiting factor in the program’s adoption for some communities, and limited financial support was also reported as a barrier for long-term implementation limiting initial adoption.

Regarding internal context, the compatibility of the telehealth fall prevention MVF program with the organization’s strategic priorities was described as important for most communities. However, organizational and structural barriers including multiple decisional levels, complex financial procedures, or limited partnerships in the community were reported as challenges. Even though financial support from Health Canada/Société Santé en français (SSF) allowed the project team to support the salary of all program facilitators and acquisition of equipment, challenges with creating effective financial procedures were raised. Many community organizations did not have the financial capacity to purchase equipment and afford salaries prior to being reimbursed, which caused important barriers.

Ongoing support and communication through the pre-implementation steps from the MVF research team was expressed as being critical for capacity building. Each community interacted with the MVF team monthly for 6–12 months before implementing the program. This may have facilitated the program adoption.

### Implementation

Four themes were identified by the program facilitators as barriers and facilitators to implementation: level of preparation and time management, interpersonal relations and telepresence, exercise facilitation and safety, and technological problem-solving.

Preparation before each class included looking back on previous weeks and introducing the week’s topic. Insufficient planning by several of the facilitators was observed by the MVF trainer in the first sessions, but only from one facilitator by week 6 demonstrating learning in this area. For time management, program facilitators initially underestimated the length of the exercise program. However, all facilitators were able to improve and deliver the entire content of each class within the planned time by week 6.

From an interpersonal relationship perspective, several facilitators showed signs of nervousness, and some were lacking in energetic disposition during the first few classes. Remote interaction with participants sometimes lacked fluidity. Some facilitators initially forgot to engage with participants virtually or were too far from the screen. However, with feedback by week 6, all facilitators were interacting with more ease, calling participants by their first names, feeling more comfortable with group discussions, and encouraging participants to talk among themselves. To stimulate motivation in participants, some program facilitators used encouragement techniques such as verbal cueing, counting repetitions out loud, and words of encouragement from the first session. Telepresence concepts such as optimal positioning in front of the screen and wearing contrasting clothing to maximize visibility were well demonstrated from the outset.

When showing exercises, a lack of clarity in the instructions occasionally caused some confusion for participants. Improvement in this area was observed at all sites during subsequent classes. Safety (i.e., wearing appropriate footwear for the exercises, correcting participants as needed, and giving advice on how to modify the exercises when some had difficulty) was well assured by program facilitators throughout the program. Infrastructure selection for community programs, including lighting, room size, and layout was adequate in most cases. Only one facilitator needed safety reminders during week 6.

Occasionally unstable internet connection and lack of technological preparation (i.e., uncharged computers, missing charging cables, and access to password-protected on-site computers) posed some challenges. Despite the occasional technological limitations, all program facilitators showed good technological problem-solving skills to ensure that all sessions could be completed smoothly. For example, several facilitators connected to the virtual session on a second device such as a laptop or tablet, placed close to them allowing them to demonstrate exercises while observing participants adequately. Program facilitators also reported that the added training and feedback received during the implementation were valuable to increase their comfort level in delivering the program.

## Discussion

The use of mixed methodologies allowed us to quantitively document strategies to reach, adopt and implementation new telehealth program for primary fall prevention while recognizing the lived experiences of program facilitators within various communities.

Since building community partnership is essential for the successful adoption of new health interventions (Mott et al., 2014), dedicating time and human resources to build these partnerships was vital to the implementation of the MVF program. Our results showed that an entire year was dedicated to reaching potential francophone communities and building partnerships. Community reach was completed by the same researcher, a physiotherapist with a PhD in rehabilitation, and expertise in telerehabilitation and fall prevention as well as lived experience animating and implementing the telehealth fall prevention program MVF.

The belief and knowledge that fall prevention should be prioritized within each organization may have facilitated adoption. Supporting the literature (Sévigny et al., 2015), results from our study show that meaningful engagement of organizations with a mission to improve access to health services for FAMC, and ongoing communication with program facilitators may have supported adoption and facilitated implementation. Organizational readiness has been documented as a key factor in the implementation of new evidence-based programs (Scaccia et al., 2015). Concordance between the mission and mandate of each organization and the aims of the MVF telehealth fall prevention program allowed for a seamless adoption of the program since community organizations were ready.

In addition to the mission of an organization, targeting the right context and demographic is critical to the adoption of a new program. An awareness of the local region is necessary for implantation to be successful. Results from our study demonstrated that program facilitators underestimated the interest for the program and acceptance rate of older adults into the program. Interestingly, two out of four provinces that adopted the program were among those with the highest rural-to-urban living proportions. This would suggest that having access to various versions of a telehealth program may facilitate recruitment for people living in rural areas. However, further efforts should be made to recruit participants from provinces where the population aged 60 and over represents more than 30% of the francophone population (British Columbia, Manitoba, Nova Scotia, and Saskatchewan).

The use of telehealth may facilitate reaching remote communities in which local French-speaking health care professionals are not available. Perspectives around technology literacy were reported by facilitators which concord with the current literature around the importance of telepresence and digital literacy (Triana et al., 2020). Experience using technology to provide health services is crucial to the success of a program. Our results demonstrated that facilitators’ skills and comfort with the technology improved over time and that the presence of the MVF trainer during the implementation was a key factor in this improvement. Since program fidelity tends to lead to successful implementation (Hill & Erickson, 2019), certain difficulties were mitigated by good preparation of the facilitators and continued education throughout the implementation. Ongoing training for the facilitators included how to demonstrate exercises while observing and providing corrections to each participant during the implementation of the program and improving their familiarity with the content. Many facilitators seemed to have underestimated the preparation required to teach the exercises with a comfortable level of confidence.

## Limitations

Our results reflect only the reach, adoption, and implementation of MVF by eight FAMC from four provinces. Future studies should focus on reaching communities from provinces and territories with a smaller pool of francophones to improve access. The financial support from SSF/Health Canada for this project allowed communities to acquire equipment, training, and support for the program implementation; therefore, transferability to other contexts when such financial support is not available may be limited. The authors are aware that the interpretation of the data may be biased as they were involved in supporting various program implementations, but their positionality statement may offer readers a better understanding of how the data was interpreted, therefore improving the trustworthiness of their interpretation.

## Implications for Practice

The shared experiences and documented strategies used by health care providers in FAMC may guide other community health care workers, clinical managers, and health care organizations in their implementation of primary prevention programs delivered using telehealth.

Time and facilitator engagement are factors well reported in the literature as essential components to successful implementation (Boothroyd et al., 2017). Results from our study support this and contribute by reporting on the importance of capacity building led by a professional with lived experience.

In addition to a formal pre-program facilitator training on the content to be delivered, ongoing workshops for all stakeholders involved in a new telehealth program should include how to use specific platforms, how to interact with your audience using technology, and how to problem-solve different scenarios and a simulation component before engaging with the participants. Follow-up supervision or training during program delivery can also be planned to solve issues specific to the program facilitator’s needs. This would enhance program fidelity.

It is important to consider the timing of implementation and alignment of the program with the strategic priorities of the organization. Assessment of an organization’s readiness should include fit between the organization’s mission and prevention program and adequate implementation timing.

## Conclusion

Networking, credibility, effectiveness, and access to financial resources are necessary strategies to facilitate the reach, adoption, and implementation of a novel telehealth primary fall prevention program. Similarly, establishing the availability of francophone human resources by training community health professionals and securing proper infrastructure and financial support before implementation is critical. Finally, using tailored reach strategies such as focusing on provinces with higher proportions of older francophone adults or rural regions where francophone health services are limited, and training local community program facilitators may lead to the successful adoption of a new telehealth fall prevention program. To ensure the sustainability of the program, a community of practice will be created to provide a space for program facilitators to exchange their experiences and ask questions.

## Appendix

**Appendix:** Complete Description of the MVF Program Reported by the 16 Items of the Consensus on Exercise Reporting Template Checklist (Slade et al., 2016)

Item category	Item description
WHAT: materials	1. Steps, TheraBand elastics, Foam squares (30 × 50 cm), Chair with armrests, computer, camera, microphone, high-speed internet connection, participants, and facilitator manual, 10 educational videos and 1 security video.
WHO: provider	2. Professionals such as a physiotherapist, occupational therapist, nurse, kinesiologist, physical activity educator, social worker, health promotion worker, or rehabilitation assistant trained by the MVF team. Each program facilitator completed a 2-day training remotely and 3-in-session follow-up during the implementation.
HOW: delivery	3. The group exercise component of the program was offered in a community center or at home through videoconference. 4. The exercises were remotely facilitated by a trained facilitator and supervised by an assistant on-site for the community version. The home-based program was not supervised. Each facilitator completed a 2-day training program that was delivered by an online physiotherapist trainer in synchronous mode. This training, which included a lecture on the content of the program, simulated presentations of information capsules and exercises, and evaluation techniques, enabled the future facilitators to acquire the necessary skills to facilitate and implement the program in a standardized manner. In addition, coaching from a certified physiotherapist and trainer during the initial implementation allowed facilitators to receive feedback on the technical, telepresence, and distance facilitation aspects of the program. Facilitators were trained using active telepresence and communication to help motivate the participants remotely. Facilitators were encouraged to engage with the participants and facilitate social interaction during the sessions. 5. Adherence was measured by recording the weekly presence of participants. At the beginning of each supervised session, the facilitator verbally asked the participants if they had completed their home-based exercises. 6. Each participant signed a consent form at the beginning of the program including details of what the participant commits to accomplish (12-week program including assessments, 10 weekly sessions, and home-based exercises). Participants have access to a calendar and manual to document their completed sessions. A pre-post assessment may motivate participants to complete the program, to improve their knowledge and physical capacity. Tools supporting the completion of exercises included paper form, videos, and demonstration by trained facilitators. Participants are encouraged to continue the exercise program and will have a follow-up 3 and 6 months after the end of the program. 7. Eligibility to the program and starting levels were established from results of the pre-assessment. Each exercise has three levels (novice, intermediate, advanced) and the program also progresses four times throughout the weeks (week 1–2, 3–5, 6–8, and 9–10). 8. Each exercise is described and illustrated in the participant’s manual. These exercises are demonstrated weekly by the trained facilitator. Participants have access to videos demonstrating the exercise program. 9. The home exercise program contains balance, mobility, lower extremity strengthening, and flexibility exercises. Participants are encouraged to observe their environments and reflect on their own behaviors which could be contributing to falls risk. 10. Ten educational videos on falls risk factors are presented, one new educational capsule per week. Overall, they touch on changes in behavior, environment, medication, health conditions, and physical activity. Participants are asked to complete a questionnaire on general knowledge regarding falls risk before and after the 10-week program. A video on “safety and how to get up from a fall” is also presented to participants. A social interaction and group discussion component is encouraged between the educational video and exercise section of each session. 11. Adverse events are documented by the program facilitator. The program facilitators have an ‘emergency file’ for each participant in case of the adverse events. Precautionary measures are taken to limit injury occurrence. Specifically, all community sessions include an in-person supervisor with CPR to be present on site. In addition, participants have a chair at arm’s reach and are positioned in the room based on their initial assessment score (novice in front, intermediate in the middle, advanced in the back). At home, participants are asked to have a phone near them when completing the program. Furthermore, training is provided to the program facilitator to manage injury risks and report adverse events on a case-by-case basis.
WHERE: location	12. Two versions of the program are offered: in community centers (max. 15 participants) and at home (max. 6 participants)
WHEN HOW MUCH: dosage	13. The exercise program consists of 4 sections for a total of 45 minutes, where sets and repetitions are different from one exercise to another. The 90-minute program is remotely delivered 1 session per week, for 10 weeks. Participants are encouraged to complete strengthening and flexibility exercises three times a week and the balance exercises daily. A) 10 minutes warm-up (3 sets of exercises completed between 5 and 30 seconds), B) 15 minutes balance (2–3 sets of exercises holding each repetition between 5 and 30 seconds), C) 10 minutes strengthening (2 sets, 12–20 repetitions or max repetitions in 30 seconds), D) 5 minutes flexibility (1 set, holding each exercise 30–60 seconds).
TAILORING: what, how	14. Three levels of difficulty and four progressions are available within the program. 15. The starting level of difficulty is tailored to each participant according to their pre-assessment total BERG Balance Scale and Five Time Sit to Stand (FTSTS) scores. Participants are not eligible if their BERG score is under 48/56 and their FTSTS is over 15 seconds. Starting levels are established from the BERG scores: Novice: 48–49, Intermediate: 50–53, advance: 54–56.
HOW WELL: planned, actual	16. A pre-post assessment allows to measure the changes in physical abilities (Berg, 5TSTS, single leg stance) and knowledge. Participants are asked to complete the weekly calendar in their manual to document what they have completed. The MVF team completes a follow-up with each trained program facilitator to ensure that the program is delivered as planned.
